# Smartphone Delivery of Cognitive Behavioral Therapy for Postintensive Care Syndrome-Family: Protocol for a Pilot Study

**DOI:** 10.2196/30813

**Published:** 2021-08-04

**Authors:** Amy B Petrinec, Joel W Hughes, Melissa D Zullo, Cindy Wilk, Richard L George

**Affiliations:** 1 College of Nursing Kent State University Kent, OH United States; 2 Department of Psychological Sciences College of Arts and Sciences Kent State University Kent, OH United States; 3 College of Public Health Kent State University Kent, OH United States; 4 Department of Surgery Summa Health Akron, OH United States

**Keywords:** postintensive care syndrome-family, mobile health app, cognitive behavioral therapy, mobile phone

## Abstract

**Background:**

Family members of critically ill patients experience symptoms of postintensive care syndrome-family (PICS-F), including anxiety, depression, and posttraumatic stress disorder. Postintensive care syndrome-family reduces the quality of life of the families of critically ill patients and may impede the recovery of such patients. Cognitive behavioral therapy has become a first-line nonpharmacological treatment of many psychological symptoms and disorders, including anxiety, depression, and posttraumatic stress. With regard to managing mild-to-moderate symptoms, the delivery of cognitive behavioral therapy via mobile technology without input from a clinician has been found to be feasible and well accepted, and its efficacy rivals that of face-to-face therapy.

**Objective:**

The purpose of our pilot study is to examine the efficacy of using a smartphone mobile health (mHealth) app to deliver cognitive behavioral therapy and diminish the severity and prevalence of PICS-F symptoms in family members of critically ill patients.

**Methods:**

For our pilot study, 60 family members of critically ill patients will be recruited. A repeated-measures longitudinal study design that involves the randomization of participants to 2 groups (the control and intervention groups) will be used. The intervention group will receive cognitive behavioral therapy, which will be delivered via a smartphone mHealth app. Bandura’s social cognitive theory and an emphasis on mental health self-efficacy form the theoretical framework of the study.

**Results:**

Recruitment for the study began in August 2020. Data collection and analysis are expected to be completed by March 2022.

**Conclusions:**

The proposed study represents a novel approach to the treatment of PICS-F symptoms and is an extension of previous work conducted by the research team. The study will be used to plan a fully powered randomized controlled trial.

**Trial Registration:**

ClinicalTrials.gov NCT04316767; https://clinicaltrials.gov/ct2/show/NCT04316767

**International Registered Report Identifier (IRRID):**

DERR1-10.2196/30813

## Introduction

### Background

Nearly 6 million patients are admitted to the intensive care unit (ICU) each year in the United States [1]. Although the majority of patients leave the ICU after a brief stay, 20% to 39% of these patients require mechanical ventilation and a potentially prolonged stay [2]. Family members of these critically ill adult patients are at risk for developing clinically significant psychological distress. Such distress is called postintensive care syndrome-family (PICS-F), and it includes symptoms of anxiety, depression, posttraumatic stress, complicated grief, and a diminished quality of life [3-10]. The prevalence of PICS-F symptoms can be as high as 69% within the first 6 months of ICU hospitalization and has been documented up to 4 years after the development of ICU illness across transitions of care to other facilities [4-6,9,11-17]. The identification and treatment of PICS-F symptoms across the continuum of recovery has been recognized and promoted by the Society of Critical Care Medicine—the leading critical care organization in the United States. Further, many experts have emphasized the need to address the gaps between transitions of care [8,18,19]. PICS-F reduces the quality of life of critically ill patients’ families and may impede the recovery of such patients [9,11,20]. Many family members of critically ill patients are called upon to provide informal caregiving during the prolonged recovery phase, which is associated with significant physical and emotional burdens [21-23]. The overall costs to society of anxiety, depression, and posttraumatic stress symptoms associated with PICS-F have not been calculated but are likely to be considerable, given the billions of dollars associated with managing the symptoms of these disorders [24-26].

A variety of strategies and interventions have been proposed to support family members during and after critical illness development, including post-ICU clinics, improved communication strategies, ICU diaries, family ICU navigators, and proactive palliative care and ethics consultation, but they have had mixed results [16,17,27,28]. A randomized controlled study of a clinician-led telephone- and web-based coping skills training program showed no improvement in psychological distress symptoms among patients and family members compared with an education program [29]. The intervention was implemented after the ICU patient and family member dyads were discharged to home, which often occurred well after the development of acute critical illness. The implementation of these clinician-led interventions requires at least moderate logistical, personnel, and financial resources and may be limited across interinstitutional transitions of care. Furthermore, family members of ICU patients are not patients themselves, and the medical services rendered to them are not currently billable. This has resulted in an inadequate medical system infrastructure for the assessment and treatment of PICS-F [10]. Therefore, effective interventions for PICS-F symptoms need to directly address the symptoms experienced by family members, be portable and longitudinal in terms of their scope for family members across transitions of care, and be made available on demand for family members while limiting the hospital resources required to implement and sustain the intervention. Strong empirical support for sustainable interventions that aim to prevent or diminish PICS-F symptoms is currently lacking.

Cognitive behavioral therapy (CBT) is a form of therapy that emphasizes cognitive and behavioral strategies for correcting unhelpful appraisals of stressful events and mitigating their influence on feelings and active coping behaviors for responding to distress [30]. CBT has become the first-line nonpharmacological treatment for the symptoms of a growing list of mental health problems, including depression, anxiety, posttraumatic stress, substance abuse, and eating disorders [31]. CBT programs delivered via mobile health (mHealth) solutions have been shown to be efficacious, cost-effective, and well accepted by individuals with mild-to-moderate symptoms of depression, anxiety, and posttraumatic stress [32-35]. Due to the development and rapid market growth of smartphone technology, mHealth apps that deliver CBT have also demonstrated significant efficacy in treating a variety of symptoms, including anxiety, depression, and posttraumatic stress [36-41]. Self-efficacy appears to be an important concept for understanding treatment gains in CBT therapy and chronic disease self-management [42-48]. Investigators have reported that the concept of self-efficacy mediates the effect between web-based and mobile CBT interventions and improvements in symptoms of stress, anxiety, and depression [49,50]. However, despite the empirical support for its effectiveness in other populations, the efficacy of delivering CBT to family members with PICS-F symptoms via mHealth technology has not been examined.

### Theoretical Framework

The theoretical basis for the proposed study is Bandura’s social cognitive theory, which describes human functioning as a reciprocal interplay among personal, behavioral, and environmental factors [51]. Perceived self-efficacy—an individual’s belief that they can perform a behavior—is a central cognitive tenet of the theory and has been identified as an important factor for explaining treatment success in CBT, the self-management of chronic conditions, and improved psychological functioning [42-48,52]. Mental health self-efficacy (MHSE)—a person’s confidence in managing his or her mental health symptoms—is a self-efficacy construct that was developed based on Bandura’s guidelines for constructing self-efficacy questionnaires and was found to be a significant mediator for the beneficial treatment outcomes of a mobile phone CBT intervention for mild-to-moderate depression, anxiety, and stress [49]. Furthermore, self-efficacy is likely to be an important factor in mobile CBT interventions with minimal therapist input, given the central role of individuals in self-monitoring and problem solving.

### Study Aims

The findings from our pilot study will allow for the collection of preliminary data that are needed for planning a fully powered randomized controlled study. The specific study aims are as follows: (1) determine the prevalence and severity of PICS-F symptoms (anxiety, depression, and posttraumatic stress), health-related quality of life (HRQOL), and MHSE in family decision makers of critically ill patients and their changes over time (at enrollment, 30 days after enrollment, and 60 days after enrollment); (2) determine differences in PICS-F symptom severity, HRQOL, and MHSE between family decision makers (ie, those of critically ill patients) receiving an mHealth app intervention and family members receiving standard care and support; and (3) determine the relationship between the dose of the mHealth app (total time spent with the app and the total number of log-ins) over the course of the study (60 days) and changes in PICS-F symptom severity (anxiety, depression, and posttraumatic stress), HRQOL, and MHSE.

## Methods

### Design

A repeated-measures longitudinal study design that involves the randomization of participants to 2 groups (the control and intervention groups) will be used. A research assistant will randomize study participants by using computer-generated random numbers after study enrollment is completed and baseline measurements are obtained.

Previous work conducted by Petrinec and colleagues [6,7,9,11] has laid the descriptive groundwork for the proposed study. The research team has recently completed a longitudinal feasibility study at Summa Health in which they examined the implementation of the mHealth app delivery of CBT to family decision makers of critically ill patients. The data from the feasibility study was used to directly inform the methodology of the proposed pilot study. The usage of the app was encouraging, and the findings of the feasibility study have been accepted for publication [53].

### Sample and Setting

The study will include family members of critically ill patients who are admitted to 1 of 2 26-bed ICUs at the Akron campus of Summa Health. A family member will be defined as a person who would be the most involved in a patient’s treatment and care decisions; the person does not need to be a blood relative. A sample size of 60 family members is planned for our pilot study (intervention group: n=30; control group: n=30). The sample size was determined according to the recommendations of Whitehead et al [54] for estimating an a priori small effect size, and an attrition rate of 30% was derived from previous studies [6,9].

### Inclusion Criteria

The inclusion criteria are as follows: (1) individuals aged 18 years or older; (2) individuals who self-identify as the family decision maker of the critically ill patient; (3) individuals who can read and speak English; (4) individuals who own a smartphone with an iOS or Android operating system; (5) family members of critically ill patients who have been in the ICU for more than 3 days; (6) family members of critically ill patients who are being mechanically ventilated and lack cognitive capacity; (7) family members of critically ill patients who are not expected to be transferred out of the ICU within 48 hours after the identification of their inclusion in the study; and (8) family members of critically ill patients aged 18 years or older. These inclusion criteria have been used in previous studies conducted by the principal investigator and other researchers [6,9,55].

### Instruments

#### Demographic Form

The information obtained from the demographic form will be collected from study participants and medical records. Data about family members’ characteristics will include demographic data, a history of treatment for psychiatric disorders (anxiety, depression, and posttraumatic stress disorder [PTSD]), a history of taking prescription medications for emotions or moods, and a history of previous ICU-related decision-making experience. Data about patients’ characteristics will include demographic data, the length of ICU stay, the duration of mechanical ventilation, admitting ICU diagnoses, baseline medical comorbidities, the baseline severity of illness, and disposition at each study time point (T).

#### Hospital Anxiety and Depression Scale

Symptoms of anxiety and depression will be assessed by using the 14-item Hospital Anxiety and Depression Scale (HADS) instrument [4,17,56,57]. The HADS is a 14-item scale with 7 items that form an anxiety subscale (HADS-A) and 7 items that form a depression subscale (HADS-D). Each of the two subscales can have scores that range from 0 to 21. Higher scores indicate higher levels of anxiety or depression symptoms. A cutoff score of ≥11 is consistent with moderate-to-severe symptoms of anxiety or depression.

#### PTSD Checklist for the Diagnostic and Statistical Manual of Mental Disorders, Fifth Edition

Symptoms of posttraumatic stress will be measured by using the 20-item PTSD Checklist for the *Diagnostic and Statistical Manual of Mental Disorders, Fifth Edition* (PCL-5) instrument [58]. The PCL-5 is a 20-item self-report measure that corresponds to the *Diagnostic and Statistical Manual of Mental Disorders,* *Fifth Edition* criteria for PTSD [59]. A total symptom severity score (range 0-80) can be obtained by summing all of the items’ scores. Higher scores indicate a higher severity of PTSD symptoms. A cutoff score of 31 or higher is the recommended indicator for a provisional diagnosis of PTSD.

#### Medical Outcomes Study 12-item Short-Form General Health Survey

The Medical Outcomes Study 12-item Short-Form General Health Survey is a 12-item self-report scale for measuring HRQOL [60,61]. Each item on the scale is scored by using a Likert-type scale. Raw scores are transformed to scores that range from 0 (worst) to 100 (best). The scale provides a summary score of 0 to 100 for the physical and mental health quality of life. Higher scores represent a more positive quality of life.

#### MHSE Scale

The MHSE Scale is a 6-item scale for measuring MHSE [49]. Each item is measured on a 10-point Likert scale that ranges from 1 (not at all confident) to 10 (totally confident). Items are summed to obtain a total score that ranges from 6 to 60. Higher scores indicate higher levels of MHSE.

### Intervention

The selection of the mHealth app was based on several criteria, as follows: (1) the app uses principles of CBT to deliver strategies for managing stress, anxiety, and depression; (2) the app is available for Android and iOS operating systems; and (3) there is a free version of the app that participants can use after the proposed study. Based on these criteria, the Sanvello (formerly known as Pacifica) app developed by Sanvello Health was chosen [62]. Sanvello has been identified as a well-designed app that is based on CBT principles, has been selected as the app of choice for other trials that have examined the implementation of the mHealth app delivery of CBT, and has been shown to be efficacious in diminishing mild-to-moderate symptoms of anxiety and depression in community samples of adults [41,63,64].

The Sanvello app is a mobile app that is marketed as a tool that provides on-demand help for managing anxiety, stress, and depression. It includes a suite of tools that are based on CBT and mindfulness principles that teach users strategies for self-managing stress, mood, anxiety, and depression. Upon initial log-in, the app asks users to select up to 3 of a possible 8 goals to work on and prompts users daily to rate their mood. Based on their mood ratings, the app suggests several activities for addressing users’ moods. The app guides users through a variety of short audio lessons or branched sessions based on their moods or goals and allows users to monitor their progress. Exercises typically take 3 to 5 minutes to complete. There is an anonymous peer support community in which users may post their thoughts and struggles as well as find listings of crisis lines and resources for users in emergency situations.

Upon randomization to the intervention group, family members will be assisted with downloading the app and creating a Sanvello account. Study participants will receive an introductory training session and instructional guidebook for reviewing basic app usage and the app’s components. Study participants will be instructed to start their mHealth app usage with the first “guided journey” module, which is called “feeling better.” The “feeling better” module has 7 individual exercises, and participants will complete 1 exercise each day. Once the entire “feeling better” module is completed, the participants will be encouraged to continue to complete the remaining “guided journey” modules—“braving anxiety,” “becoming mindful,” “taking control,” and “building confidence.” These remaining modules are composed of 6 to 11 individual exercises. Additionally, participants will be allowed to use the components of the mHealth app in whichever way they want. This includes accessing tools that allow users to track their exercise and sleep habits; communicating with web-based coaches; and sharing experiences in a nonjudgmental, web-based, and secure forum. Participants will receive weekly text reminders that encourage them to use the mHealth app. At the conclusion of the study, app usage data (the number of log-ins, time spent with the app, etc) will be collected and provided to the research team by Sanvello via an encrypted, password-protected, and deidentified data file.

### Recruitment

The research assistant will visit the ICU 3 times per week to identify newly admitted patients who are eligible for the study. The research assistant will screen for eligibility by using medical records and consulting with the health care team. If the eligibility criteria are met by a family member, they will be approached for enrollment within the first week after patient admission. The research assistant will consult with the bedside nurse to identify eligible family members while visiting patients or will contact eligible family members by phone. Family members will be randomized to the intervention group or control group after enrollment and data collection at enrollment are complete.

The longitudinal study design has 3 data collection points. Data will be collected by using the same methods as those in a previous study conducted by Petrinec and Martin [9] upon enrollment into the study (T1), 30 days after study enrollment (T2), and 60 days after study enrollment (T3). The data collection process for each time point is shown in Table 1. At 30 and 60 days after study enrollment, family members will be contacted by phone, email, or standard mail. Participants’ preferences for follow-ups will be identified upon their enrollment into the study. A US $30 gift card will be offered at T1 and T2, and a US $50 gift card will be offered during follow-up data collection in T3 for a possible total of US $110.

**Table 1:** Data collection checklist.

Data collected	T1^a^ (Enrollment)	T2^b^ (30 days after enrollment)	T3^c^ (60 days after enrollment)
Demographic data	✓	✓	✓
Hospital Anxiety and Depression Scale score	✓	✓	✓
PCL-5^d^ score	✓	✓	✓
Mental Health Self-Efficacy Scale score	✓	✓	✓
SF-12^e^	✓		✓
Mobile health app usage			✓

^a^T1: time point 1.

^b^T2: time point 2.

^c^T3: time point 3.

^d^PCL-5: Posttraumatic Stress Disorder Checklist for the *Diagnostic and Statistical Manual of Mental Disorders, Fifth Edition.*

^e^SF-12: Medical Outcomes Study 12-item Short-Form General Health Survey.

### Data Analysis and Statistical Plan

Data will be analyzed by using SPSS, version 25 (IBM Corporation), and descriptive statistics will be used to assess the frequencies and variability of the data, coding inaccuracies, outliers, and missing data. The statistical plan for each specific aim is detailed in the following subsections.

#### Aim 1

We will report study variables with descriptive statistics. Changes over time will be examined with a repeated-measures analysis of variance.

#### Aim 2

Differences between the intervention and control groups will be assessed with a two-tailed Student *t* test. Differences between groups with regard to the severity of PICS-F symptoms will be used to calculate the effect size for the intervention.

#### Aim 3

The relationship between total mHealth app doses and longitudinal changes in study variables will be examined with a Pearson correlation analysis.

### Human Subjects and Ethical Issues

The study will undergo review and approval by the institutional review board at Summa Health. The investigators have considerable experience in conducting research on individuals with symptoms of anxiety, depression, and posttraumatic stress. The study will present no more than minimal psychological risk and harm, which largely come from the possibility that answering the questions on the anxiety, depression, and stress instruments may be distressing for participants. Psychological risk will be minimized by emphasizing that study participants can stop participating in the study at any time and are not obligated to answer any question that they find to be distressing. Family members who exhibit clinically significant symptoms will be referred to their primary care physician. Family members of patients who die during the study will be referred to local bereavement support groups.

There will be a low risk of privacy or confidentiality loss. This risk will be minimized via the following measures: (1) the only record linking participants and the research data will be the consent document; (2) consent documents will be kept in a locked cabinet in the locked office of the principal investigator; (3) data will be entered and stored by the research assistant on REDCap (Research Electronic Data Capture; Vanderbilt University)—a secure, Health Insurance Portability and Accountability Act–compliant, web-based platform; (4) all data files obtained for analysis will be stored on a password-protected laptop computer, which will be stored in a locked room; and (5) the principal investigator, coinvestigators, and research assistant are the only individuals who will have access to the data files.

## Results

Recruitment for our pilot study began in August 2020. During recruitment, challenges arose due to the COVID-19 pandemic. There was a short period of time when research studies at Summa were placed on hiatus due to COVID-19–related restrictions. This was followed by challenges in recruiting family members while limitations were placed on family visitation to the hospital. Despite these challenges, data collection and analysis are expected to be completed by March 2022. The dissemination of our findings will be accomplished through conferences and publications.

## Discussion

The use of a self-care mHealth app represents a novel approach to addressing the needs and untoward psychological symptoms experienced by family members of critically ill patients by leveraging technology and the growing market penetrance of smartphone ownership. The proposed study emphasizes self-care, which can function as an adjunct and complement to existing nursing efforts for supporting family members of critically ill patients without significantly increasing the need for care delivery resources. Importantly, the proposed mHealth app offers a portable, “just-in-time” benefit to users by being available to them on demand via their smartphone across transitions of care. The findings of our study will inform the planning and implementation of a randomized controlled trial. Additionally, the findings may help to direct collaborations with app developers for modifying existing apps or creating new apps that emphasize self-care specifically for family members and caregivers of acutely and chronically ill patients.

## Appendix

Multimedia Appendix 1 Peer review report from the American Association of Critical-Care Nurses.

## Abbreviations

CBT: cognitive behavioral therapy
HADS: Hospital Anxiety and Depression Scale
HRQOL: health-related quality of life
ICU: intensive care unit
mHealth: mobile health
MHSE: mental health self-efficacy
PCL-5: Posttraumatic Stress Disorder Checklist for the Diagnostic and Statistical Manual of Mental Disorders, Fifth Edition
PICS-F: postintensive care syndrome-family
PTSD: posttraumatic stress disorder
REDCap: Research Electronic Data Capture
