# Supplementary material for: Smartphone Delivery of Cognitive Behavioral Therapy for Postintensive Care Syndrome-Family: Protocol for a Pilot Study
Source: JMIR Res Protoc. 2021 Aug 4;10(8):e30813. doi: 10.2196/30813 (PMC8374657; doi:10.2196/30813)
Supplement: Multimedia Appendix 1 [file resprot_v10i8e30813_app1.pdf]

Applicant Last Name:	First Name:	Comments and feedback to applicant regarding aims	Comments and feedback to applicant regarding background and significance	Comments and feedback to applicant regarding methodology	Comments and feedback to applicant regarding ethical considerations	Comments and feedback to applicant regarding feasibility	Indicate the rationale for your recommendation and required changes, if applicable, below.	Additional feedback to applicant
Petrinec	Amy						This is a great study. PICC is becoming widely recognized but not so much for the family. Using technology for this is a great idea moving forward and may lead additional uses of technology for similar populations.	
Petrinec	Amy			"those with clinical significant symptoms will be referred to PCP"- How and at what point in study intervention will this be completed? Stated there are no links to data and it will be coded.	Concern for recognition and appropriate escalation of symptoms.		Reasonable budget requests. Question mileage and parking for investigators.	
Petrinec	Amy						What a terrific idea to study an intervention for PICS-F	This is a terrific proposal, well done. Great area of research, glad you are exploring an intervention.
Petrinec	Amy	objectives and hypotheses are well matched		- unclear what happens if the patient dies in the ICU -method of randomization not described - some clinical data that are collected are not justified or discussed in the methods section		the project is currently funded. Unclear what is the added benefit to the proposed study- what is the difference between this study and the funded study?	- the study is already funded- what is the added benefit of the current proposal? How is this study different from the funded study? - how does the study take into account family members of patients that died either in the ICU or soon thereafter?	
Petrinec	Amy	Specific hypotheses not stated. The duration of data collection (60 days) seems short for a 2 year study	Excellent review of literature and use of a theoretical framework	MIssing information on tool reliability and validity. What does the control group receive for standard care?			Very important topic, well-done proposal. I am a little concerned about the short follow-up duration and sample size.	Excellent proposal for a highly relevant study. A few weaknesses-lack of description of what standard care entails, short end point for a 2 year study-please consider obtaining measures at 90 or 180 days. If you have expected attrition, you will end up with only 24 subjects in each group.
